# Lateral sinus thrombosis and brain abscess as a complication of cholesteatoma

**DOI:** 10.1016/S1808-8694(15)30850-8

**Published:** 2015-10-18

**Authors:** João Alcides Miranda, Lucila Lahan Martins, Marcello Henrique de Carvalho Borges, Nelson Solcia Filho, Elaine de Abreu Mendes

**Affiliations:** 1 Otorhinolaryngologist, Hospital Sociedade Beneficente São Camilo, Caxambu - MG.; 2 Physician, otorhinolaryngology resident, Instituto Penido Burnier.; 3 Physician, resident in the Otorhinolaryngology Department, Hospital Universitário São Francisco.; 4 Otorhinolaryngologist, assistant physician in the Otorhinolaryngology Department, Hospital Universitário São Francisco.; 5 Doctor in Otorhinolaryngology, USP. Head of the Otorhinolaryngology Department, Hospital Universitário São Francisco.; Hospital Universitário São Francisco, Bragança Paulista - SP.

**Keywords:** brain abscess, otitis media, lateral sinus thrombosis

## INTRODUCTION

Intracranial complications of otitis media are still a condition of risk with a high mortality rate, notwithstanding the significant reduction of such complication with the advent of antibiotics.^1^, ^2^

Although less frequent, chronic cholesteatomatous otitis media is usually associated with the highest risk of complications, given its destructive and invasive potential. These complications may be subdivided didactically into temporal and extratemporal conditions.^2^, ^3^

The most common intracranial complications include: meningitis, cerebral abscess and lateral sinus thrombosis,^1^ the latter being present in 5 to 18,3% of intracranial complications due to otitis.^3^ Computed tomography and magnetic resonance imaging provide an early diagnosis and may detect other intracranial complications. Aggressive surgical therapy is mandatory.^4^

## CASE STUDY

**Figure 1:**
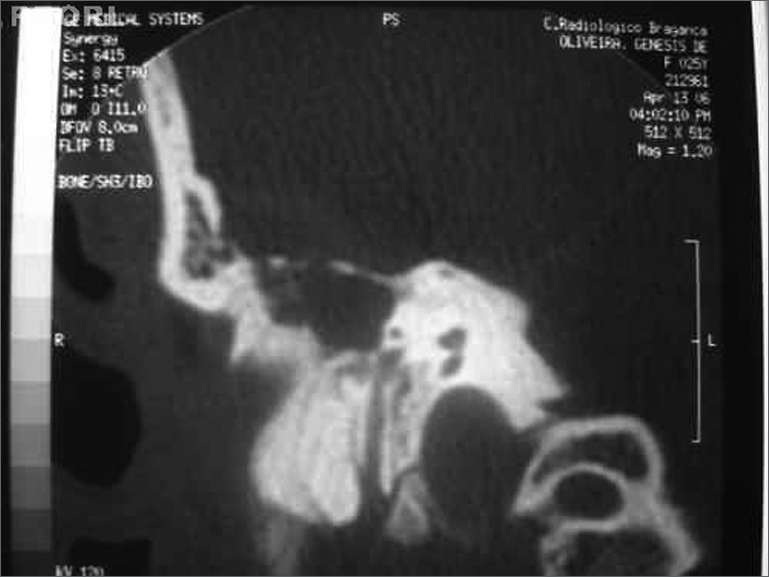
Computed tomography of the right ear (coronal section), showing erosion of the tegmen tympani.

GO, a female patient aged 25 years, had been admitted 20 days before into another hospital, presenting right otorrhea and intense holocranial headache. She was referred to our unit due to respiratory distress. The physical exam showed signs of septicemia, requiring orotracheal intubation. Otoscopy revealed a yellowish fetid secretion in the right ear. Antibiotic therapy with ceftriaxone and clindamycin was initiated based on a diagnosis of otitis media with complications. Otorhinolaryngological and neurosurgical assessments were made, and a computed tomography of the cranium and temporal bones was requested. These exams revealed significant opacification of the right middle ear and erosion of the tegmen tympani (Fig. 1), a cerebral abscess and thrombosis of the right lateral sinus. Surgical exploration of the right ear following clinical stabilization was indicated; the Neurosurgeon decided for a conservative treatment.

Doppler ultrasound of the neck showed that the lumen of the internal right jugular vein was increased and contained hyperechoic material suggesting a thrombus, which extended from the cranial base until its junction with the subclavian vein, which discarded the possibility of ligature of the jugular vein. The patient was submitted to right radical mastoidectomy, showing exposure of the sigmoid sinus; material suggesting a cholesteatoma was removed, and this diagnosis was confirmed lateral by histopathology. The patient was discharged from hospital on the third postoperative day, and progressed uneventfully.

## DISCUSSION

Intracranial complications of otitis media have decreased with the advent of antibiotics; the incidence has fallen from 2.3% to 0.04%.^2^ Currently, lateral sinus thrombosis is an otological complication occurring in teenagers and young adults; the otogenic cerebral abscess remains a severe and potentially lethal condition.^2^, ^3^ Our patient’s clinical picture suggested chronic cholesteatomatous otitis media complicated by lateral sinus thrombosis. A cerebral abscess was found when investigating concomitant intracranial complications. A clinical diagnosis should be confirmed by imaging methods.^2^

Anticoagulant heparin therapy is controversial; some authors defend its use, while others find it unnecessary in the treatment of lateral sinus thrombosis, fearing rupture of the thrombus and septic dissemination.^3^

The surgical procedure is radical mastoidectomy with removal of diseased material and full opening of the sigmoid sinus into the mastoid cavity. Some authors recommend intervening on the lateral sinus and the internal jugular vein.^2^, ^3^ We decided not to touch these structures, since they had been shown to be completely occluded in Doppler ultrasound.

It is important to have a multidisciplinary approach when treating these complications.

## FINAL COMMENTS

The mortality and morbidity of the complications of otitis remain high, especially in cases with intracranial involvement. A high degree of clinical suspicion is essential for a timely diagnosis and management of these complications.
